# Walking 200 min per day keeps the bariatric surgeon away

**DOI:** 10.1016/j.heliyon.2023.e16556

**Published:** 2023-05-22

**Authors:** Daniel H. Pfaff, Gernot Poschet, Rüdiger Hell, Julia Szendrödi, Aurelio A. Teleman

**Affiliations:** a German Cancer Research Center (DKFZ), 69120 Heidelberg, Germany; b Department of Internal Medicine I and Clinical Chemistry, Heidelberg University Hospital, 69120 Heidelberg, Germany; c Centre for Organismal Studies (COS), Heidelberg University, 69120 Heidelberg, Germany; d Heidelberg University, 69120 Heidelberg, Germany

**Keywords:** Metabolome, Physical activity, Exercise, Gut leakiness, Exerkine, Exerkinome, Walking, Weight loss intervention

## Abstract

Exercise and increased physical activity are vital components of the standard treatment guidelines for many chronic diseases such as diabetes, obesity and cardiovascular disease. Although strenuous exercise cannot be recommended to people with numerous chronic conditions, walking is something most people can perform. In comparison to high-intensity training, the metabolic consequences of low-intensity walking have been less well studied. We present here a feasibility study of a subject who performed an exercise intervention of low-intensity, non-fatiguing walking on a deskmill/treadmill for 200 min daily, approximately the average time a German spends watching television per day. This low-impact physical activity has the advantages that it can be done while performing other tasks such as reading or watching TV, and it can be recommended to obese patients or patients with heart disease. We find that this intervention led to substantial weight loss, comparable to that of bariatric surgery. To study the metabolic changes caused by this intervention, we performed an in-depth metabolomic profiling of the blood both directly after walking to assess the acute changes, as well as 1.5 days after physical activity to identify the long-term effects that persist. We find changes in acylcarnitine levels suggesting that walking activates fatty acid beta oxidation, and that this mitochondrial reprogramming is still visible 1.5 days post-walking. We also find that walking mildly increases gut permeability, leading to increased exposure of the blood to metabolites from the gut microbiome. Overall, these data provide a starting point for designing future intervention studies with larger cohorts.

## 1 Introduction

Exercise and increased physical activity are vital components of the standard treatment guidelines for chronic diseases such as diabetes mellitus [1], obesity [2] and cardiovascular disease [3,4]. In some cases, strenuous exercise can have, however, a negative impact on cardiovascular health [[5], [6], [7], [8], [9]]. Furthermore, strenuous exercise cannot be recommended to patients that are obese or have cardiovascular disease. In terms of weight loss, standard exercise regimens often lead to mild benefits, and a yo-yo effect whereby the weight is quickly regained once the intervention is stopped [10]. Nonetheless, diabetic and prediabetic patients benefit from mild physical activity, with a measurable decrease in cardiovascular mortality for every 2,000 steps of additional walking per day, prompting the American Diabetes Association to recommend an optimum of 10,000 steps per day in their guidelines [1,11]. Hence, there is a need to find a regimen of mild physical activity that can cause weight loss, and to understand the metabolic consequences of walking.

We describe here a feasibility study where we implement walking at a desk/treadmill combination (‘deskmill’) as a low-intensity physical activity that explicitly avoids exhaustion. This low intensity allows for the intervention to be performed for an extended period of time, thereby compensating for the low intensity with a higher intervention duration. Since the average German watches 220 min television per day [12] our intervention consists of 200 min of walking at the deskmill per day, 6 days per week. Due to the low intensity of this intervention, it can be performed simultaneously with other activities such as watching television or reading. This aims to overcome the problem that exhaustive exercise (e.g. 1 h 3 times per week) can still allow a person to remain sedentary 97% of their awake time per week. Often exercise interventions are accompanied by lifestyle interventions aimed at changing the participants’ diets [13]. To avoid any confounding effects caused by nutrition, however, no changes to the diet were implemented here, neither in overall amount nor composition. This was done with the aim of avoiding caloric restriction-induced muscle catabolism [14], which can counteract some of the beneficial effects of exercise [15]. Furthermore, it emulates a real-world case where a subject would undertake such an intervention. As a first step towards testing this on a larger cohort of participants we report here a feasibility study where we perform an analysis of one subject with frequent sampling, which can be used to guide the design of future studies with larger participant numbers.

The molecular pathways that confer health benefits in response to physical activity, and in particular walking, remain poorly understood [16]. These are likely mediated in part by “exerkines” - factors secreted in response to physical activity into the bloodstream [17]. Exerkines including metabolites [18,19], cytokines [20], lipids [21], exosomes [22,23] and nucleic acids such as microRNAs [24] have now been shown to respond to exercise and to originate from a variety of tissues [16]. For instance, IL-6 was identified in 2000 as the first exerkine originating from muscle [25].

Most exerkines have been identified in response to intensive exercise. These are relevant, given that the American Heart Association recommends 150 min of moderate or 75 min of vigorous exercise per week for the general population [3]. In comparison, however, the impact of mild physical activity such as walking on the exerkinome is less well understood. Hence, to characterize the metabolic consequences of walking, and to profile the metabolite-fraction of the exerkinome in response to walking, we perform here an in-depth metabolomic analysis of the subject’s serum before walking, directly after walking, and 1.5 days after walking. Similar to the currently ongoing study of the Molecular Transducers of Physical Activity Consortium (MoTrPAC; NCT03960827) [26], we study both the acute changes observed in the blood metabolome directly after physical activity, as well as the chronic changes that persist 1.5 days after the activity. We aimed to sample frequently, yielding a thorough investigation of the subject’s blood metabolome which can be used as a reference for designing future intervention trials with larger participant numbers but lower sampling rates.

## 2 Materials and methods

### 2.1 Participant characterization

The study design was approved by the local ethics committee (Ethics Committee of the Medical Faculty of Heidelberg: Trial Code Number S-518/2022). The study was performed on a single obese but otherwise healthy 37-year-old male participant, who provided written informed consent. His height was 180 cm and his body weight 117.6 kg (BMI 36.3 kg/m^2^) at the start of the study.

No regular medication was taken before enrolment. To avoid gallstone formation due to a change in bile acid composition after rapid weight loss, a low dose of 250 mg of ursodeoxycholic acid was taken on every day of walking. Hence all baseline samples, both before and during the intervention, are without ursodeoxycholic acid treatment for at least 1.5 days. The participant led a sedentary lifestyle 12 months prior to the start of the study, with the body weight being stable for at least 6 months. No weight loss interventions such as changes in the diet were applied during the study or within 12 months before the study. Nutrition was consumed until satiety was reached. Diet was not recorded. Meals were composed of a normal German diet, while in the mornings a yoghurt with cereals was consumed as a standard breakfast. The subject was asked weekly to confirm that his diet during the intervention was unchanged compared to his prior diet.

### 2.2 Experimental design

Using Sarstedt 23G Safety-Multifly®-Needles blood was drawn to fill two Sarstedt S-Monovette® 7.5 ml serum tubes in the mornings of day −2, day −1 and day 0 at 07:30 a.m. The last meal was consumed at 8:00 p.m. the evening before. After blood was drawn and body weight recorded using a scale, the standard breakfast and the ursodeoxycholic acid were ingested. The vials with the coagulated blood were centrifuged at room temperature for 15 min at 1500 rpm, then the serum was aliquoted into 1.5 ml Eppendorf tubes, snap-frozen in liquid nitrogen and stored at −80 °C until further analysis.

At 10:15 a.m. on day 0 the participant started walking on the deskmill (Maxxus TX910 treadmill paired with a customized desk) at a speed that was slow enough for the subject to comfortably maintain it for 200 min without break (starting 2.5 km/h). Heart rate was not monitored. Water without additives was consumed ad libitum while walking. Directly after finishing the 200 min walking, blood was drawn again to fill 2 serum tubes, which were then processed in the same way as previously described to obtain the first post-walking sample. During the following 5 days the 200 min walking workouts were repeated in the evenings. Day 6 was designated as a resting day. On day 7 the first trained baseline sample was obtained as previously described. The last walking phase had been concluded 36h prior to sample collection. On day 7 the second post-walking sample was generated as previously described. This setup was repeated for 4 weeks with weekly sample collection after 36h of rest until day 28, after which the experiment was concluded. No adverse events were reported during this study. Every 200 min walking bout was performed as scheduled.

### 2.3 Metabolomics analysis

In total, 630 metabolites, covering 14 small molecule and 12 different lipid classes, were analyzed using the MxP Quant 500 kit (Biocrates) following the manufacturer’s protocol. In brief, 10 μl serum were pipetted into a 96 well-plate containing internal standards and dried under a nitrogen stream using a positive pressure manifold (Waters). 50 μl of a 5% phenyl isothiocyanate (PITC) solution were added to each well to derivatize amino acids and biogenic amines. After 1h incubation time at room temperature, the plate was dried again. To extract the metabolites, 300 μl 5 mM ammonium acetate in methanol were pipetted to each filter and incubated for 30 min. The extract was eluted into a new 96-well plate using positive pressure. For further LC-MS/MS analyses, 150 μl of the extract was diluted with an equal volume of water. For FIA-MS/MS analyses, 10 μl extract was diluted with 490 μl of FIA solvent (provided by Biocrates). After dilution, LC-MS/MS and FIA-MS/MS measurements were performed. For chromatographical separation, an UPLC I-class PLUS (Waters) system was used coupled to a SCIEX QTRAP 6500+ mass spectrometry system in electrospray ionization (ESI) mode. Data were analyzed using the Analyst (Sciex) software suite and transferred to the MetIDQ software (Biocrates) which was used for further data processing and analysis. All metabolites were identified using isotopically-labeled internal standards and multiple reaction monitoring (MRM) using optimized MS conditions as provided by Biocrates. For quantification, either a seven point calibration curve or one point calibration was used depending on the metabolite class.

### 2.4 iFABP2 ELISA

Serum samples were analyzed for gut permeability by using a Human FABP2/Fatty Acid-Binding Protein, Intestinal ELISA Kit (Sigma #RAB0537) according to the manufacturer’s instructions. Initially a dilution series of the provided standard using assay diluent C was prepared. Wash Buffer was diluted with ddH2O to 1X and brought to room temperature the day of the experiment. Serum samples were taken from −80 °C storage, thawed and mixed well without vortexing. The thawed serum was diluted 1:2 in Assay diluent C, then 100 μl of diluted serum, 100 μl of Assay diluent C as blank or 100 μl of standard dilutions were pipetted to the provided microplate wells in duplicates. The microplate was covered and incubated for 2.5h with gentle shaking at room temperature. The well contents were discarded by inverting the microplate and blotting it against paper towels to completely remove any liquids. Then four washing steps with 300 μl of Wash Buffer were performed. After preparing a 80X biotin concentrate using 1X Assay Diluent B and the provided biotin conjugate, 100 μl of said concentrate were added to the wells and incubated for 1h at room temperature with gentle shaking. Afterwards the contents were removed and 4 washing steps using Wash Buffer were performed. Then the provided Streptavidin-HRP was diluted 1,500-fold with 1X Assay Diluent B. Of the diluted Streptavidin-HRP solution 100 μl were added to each well and incubated for 45 min at room temperature with gentle shaking. Again, well contents were removed and washed 4 times. Then 100 μl of TMB Substrate were added to each well and the microplate was incubated for 30 min at RT in the dark with gentle shaking. At the end 50 μl of Stop Solution were added to each well and gently mixed by tapping the side of the plate gently. OD450 was recorded within 30 min of Stop Solution addition. After subtracting blank absorption values, linear regression was applied to the samples in the linear range of the absorption curved and was then used to calculate the iFABP-2 concentration in the serum samples after correcting for the appropriate dilution factor.

### 2.5 Quantifications and statistical analysis

Statistical analysis was performed using Microsoft® Excel for Mac and GraphPad Prism. Graphs were generated using GraphPad Prism and compiled into figures using Affinity Photo. The metabolite heatmap was generated using MathWorks® MATLAB’s Clustergram function. For Fig. 2A and B and Fig. 5E, statistical significance was calculated by grouping samples (e.g. baseline or post-walking) and comparing groups using unpaired two-sided t-tests. For Fig. 2C–D, a regression *t*-test was performed on the slope of the data points to test for difference from zero. In both cases, a p-value of 0.05 was used as a cutoff to determine significance. Details provided in figure legends.

## 3 Results

### 3.1 200 min of daily walking is aerobic and not stressful, but strongly affects weight, fitness and the metabolome

**Fig. 1:**
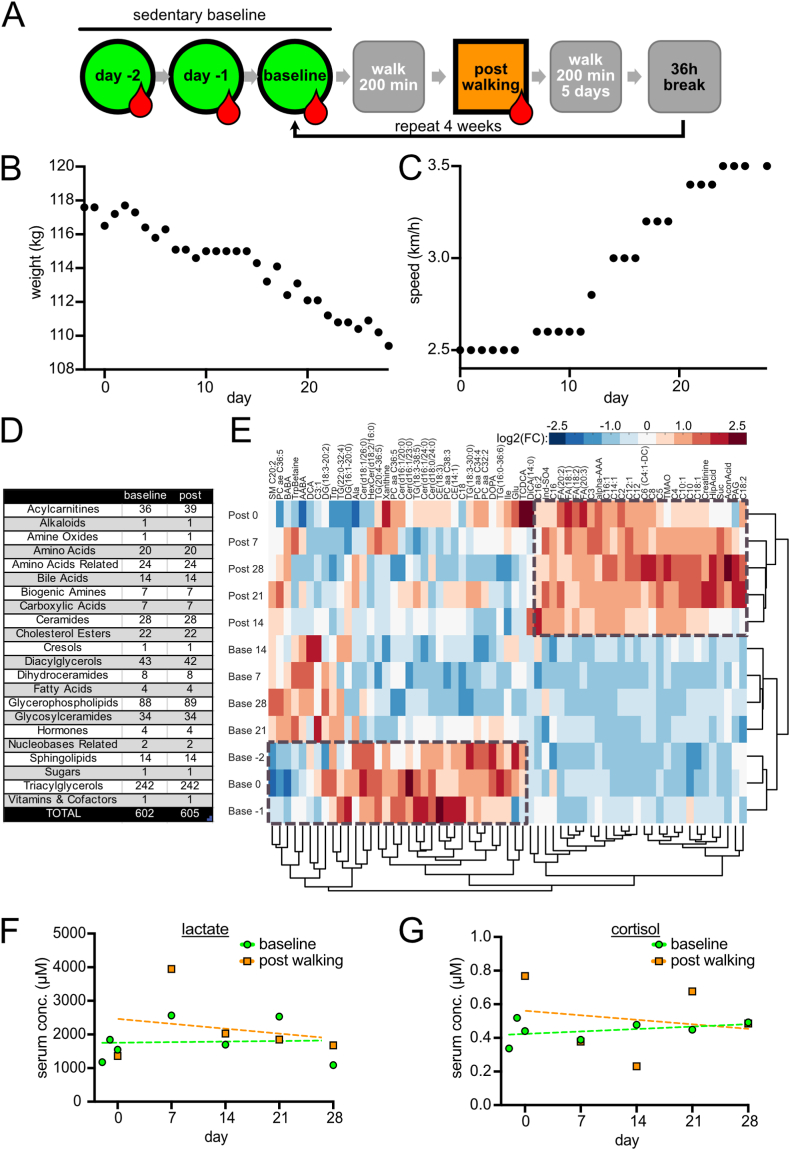
Experimental setup and characteristics (A) Schematic diagram illustrating the experimental design of the study. Blood drops symbolize blood sampling. (B) Weight curve showing a mostly linear decrease in body weight as measured daily. (C) Walking speed as recorded daily. The speed was increased when at the end of the 200 min walking bout another 100 min of walking would have subjectively considered possible without reaching fatigue. (D) Table displaying the detected metabolites grouped by their respective substance classes. (E) Heatmap shows levels of the metabolites which change significantly (full analyses shown in Fig. 2A–D). Displayed is the log2 (fold change) for each metabolite relative to the average value for that metabolite across all samples. Samples and metabolites are clustered by unsupervised hierarchical clustering. (F) Stable lactate levels show that the intervention was performed aerobically. (G) Stable cortisol levels show the intervention was not registered as stressful by the body.

The intervention was performed by a single participant with stable body weight 3 months prior to the start of the study (Suppl. Fig. 1), who led a sedentary lifestyle without regular exercise or sports. We obtained untrained baseline serum samples by drawing blood on days −2, −1 and 0 relative to the start of the intervention, always at 7:30 in the morning after overnight fasting (Fig. 1A). The serum was centrifuged, aliquoted and frozen. The participant walked 200 min on a deskmill at a speed slow enough to not cause noticeable fatigue (initially 2.5 km/h). Then the first post-walking serum sample was collected. In the following 5 days the participant walked 200 min on the deskmill in the evening, then rested on day 6. In the morning of day 7, 36h after the last bout of walking, the experimental setup of day 0 was repeated, collecting first a baseline sample, then walking, then collecting a post-walking sample. The intervention continued for 4 weeks with sample collections at days 14, 21, and 28, each time collecting the baseline samples after 36 h of rest. The body weight was measured daily and showed a rapid weight loss of 8.2 kg within 4 weeks (Fig. 1B, Suppl. Fig. 1). The intervention started with a rather slow walking speed of 2.5 km/h and was adjusted by +0.1 km/h by the participant whenever he felt that he could continue the intervention for another hour without reaching the point of fatigue. By the end of the experiment, the participant reached 3.5 km/h (Fig. 1C), suggesting an increase in muscle volume and/or cardiorespiratory fitness, that we did not measure. We then analyzed the metabolome of all 12 serum samples (3 ‘untrained’ baselines prior to the intervention, 4 ‘trained’ baselines during the intervention, and 5 post-walking samples) using a Biocrates MxP Quant 500 kit which enables reproducible, absolute quantification of up to 630 metabolites.

**Fig. 2:**
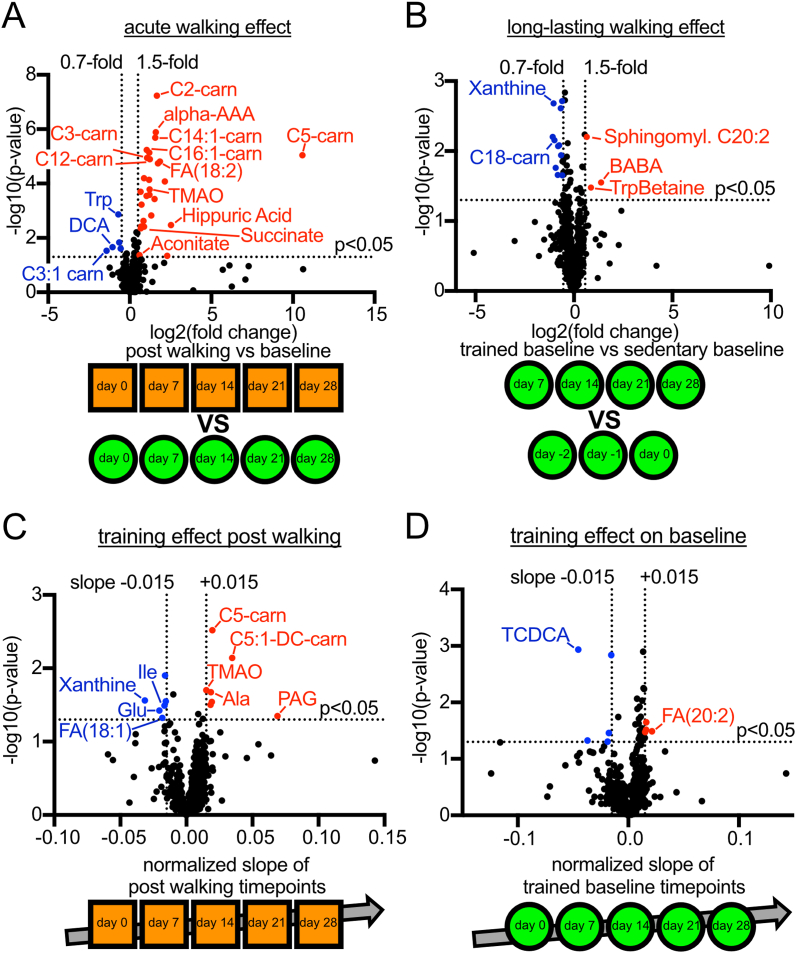
Statistical analyses of the metabolomics dataset (A) Volcano plot showing the acute effect of walking on metabolite levels by directly comparing the baseline samples to their corresponding post-walking samples (unpaired two-sided t-tests, p < 0.05). A 1.5-fold (log2(FC) = 0.59) increase or decrease of each group’s average was applied as a cutoff to define relevance. Acylcarnitines are annotated as “CX-carn” with X being the acyl chain length. (B) Volcano plot showing the chronic effect of walking on metabolite levels by comparing the trained baseline samples to the untrained baseline samples (unpaired two-sided t-tests, p < 0.05). A 1.5-fold (log2(FC) = 0.59) increase or decrease of each group’s average was applied as a cutoff to define relevance. (C) Volcano plot of the training effect on post-walking samples, calculated as the slope of the post-walking samples over time (*t*-test of the regression coefficient, p < 0.05). A slope of ± 0.015 was applied as a cutoff to define relevance. (D) Volcano plot of the training effect on baseline samples, calculated as the slope of the baseline samples over time (*t*-test of the regression coefficient, p < 0.05), starting from the last untrained baseline sample. A slope of ± 0.015 was applied as a cutoff to define relevance.

In total, we detected 605 metabolites (Fig. 1D, raw data in Table S1). Of these, 602 were present in both baseline and post-walking samples, whereas 3 metabolites (acylcarnitines) were exclusively detected in the post-walking samples. Fig. 1E shows a heatmap of the metabolites that change significantly between samples. Both hierarchical clustering and visual inspection of the heatmap shows that the three categories of samples – untrained baseline samples, trained baseline samples, and post-walking samples – are clearly distinguishable from one another (grey boxes, Fig. 1E). Interestingly, this indicates that even 1.5 days after the training period (‘trained’ baseline) the subject’s metabolism has not returned to the pre-intervention state. As expected, lactate levels remain unchanged when comparing baseline to post-walking samples, indicating that the physical activity was performed aerobically (Fig. 1F). Although cortisol levels fluctuated between samples, they did not show a consistent increase post-walking and were within normal physiological range, suggesting that the exercise performed was not stressful (Fig. 1G).

### 3.2 Some metabolites show an increase post-walking, which also increases with training

We analyzed our metabolomics dataset from several different perspectives. We present here a general overview of the metabolic changes we observed, and will analyze individual metabolites more in-depth further below. We first asked whether walking caused an acute change in the metabolome by comparing all post-walking samples to the corresponding baseline samples (Fig. 2A). Since 200 min of physical activity causes a substantial change in the physiological state of the body, we expected this comparison to reveal many metabolic changes. Indeed, this analysis yielded 32 metabolites that show a significant change in their serum concentration (p < 0.05 and |log2(FC)|>0.59, Fig. 2A). A full list is provided in Table S2. Amongst these, more metabolites increase than decrease in concentration after physical activity. The most prominent changes involve short and medium-chain acyl-carnitines, indicative of elevated fatty acid beta-oxidation (more below). The only amino acid we found changing is tryptophan, which decreases. The drop in the bile acid Deoxycholic acid (DCA) was expected, because we administered to the subject during the intervention ursodeoxycholic acid, which promotes bile secretion, to prevent possible gallstone formation from rapid weight loss.

As a second analysis, we asked whether the intervention caused a longer-lasting shift in the subject’s metabolic profile by comparing the trained baseline samples collected during the 4-week intervention to the untrained baseline samples collected at days −2, −1 and 0 before the start of the intervention (Fig. 2B). Any changes found here reflect physiological shifts that last longer than 36-h after walking. Interestingly, we found 11 metabolites which showed a significant decrease, such as C18:0-carnitine, and 3 which showed an increase, such as the aminobutyrates BABA and AABA (p < 0.05 and |log2(FC)|>0.59, full list in Table S3, Fig. 2B). Overall, the long-lasting changes between the trained and untrained baseline samples were less pronounced than the acute changes between baseline and post-walking samples. As discussed more in depth below, this suggests, nonetheless, that walking induces physiological changes that last longer than 1.5 days.

As a third analysis, we asked whether there was a training effect of the intervention – i.e. whether there were any metabolites that progressively increased or decreased over the 4-week duration of the intervention. We performed this analysis separately for the post-walking samples (Fig. 2C) and the baseline samples (Fig. 2D). A time-dependent trend in the post-walking samples indicates that the body is responding differently to the very same physical activity (200 min walking) as a consequence of the training, whereas a trend in the baseline samples indicates that the training also has cumulative effects on the resting physiology of the subject. Interestingly, we found 7 and 6 metabolites that increase or decrease over time, respectively, in the post-walking samples (Table S4) and 4 and 5 metabolites that increase or decrease over time, respectively, in the baseline samples (Table S5). The metabolites that respond to training are often also hits when comparing post-walking samples to baseline samples, such as C5-carnitine and TMAO (Fig. 2A). Some of the other metabolites that change are amino acids (Fig. 2C). Very few metabolites, however, show a significant training effect when comparing the baseline samples over time (Fig. 2D). As for DCA, the drop in the bile acid Taurochenodeoxycholic acid (TCDCA) is likely due to the ursodeoxycholic acid we administered.

### 3.3 Walking increases baseline and post-walking fatty acid beta-oxidation

**Fig. 3:**
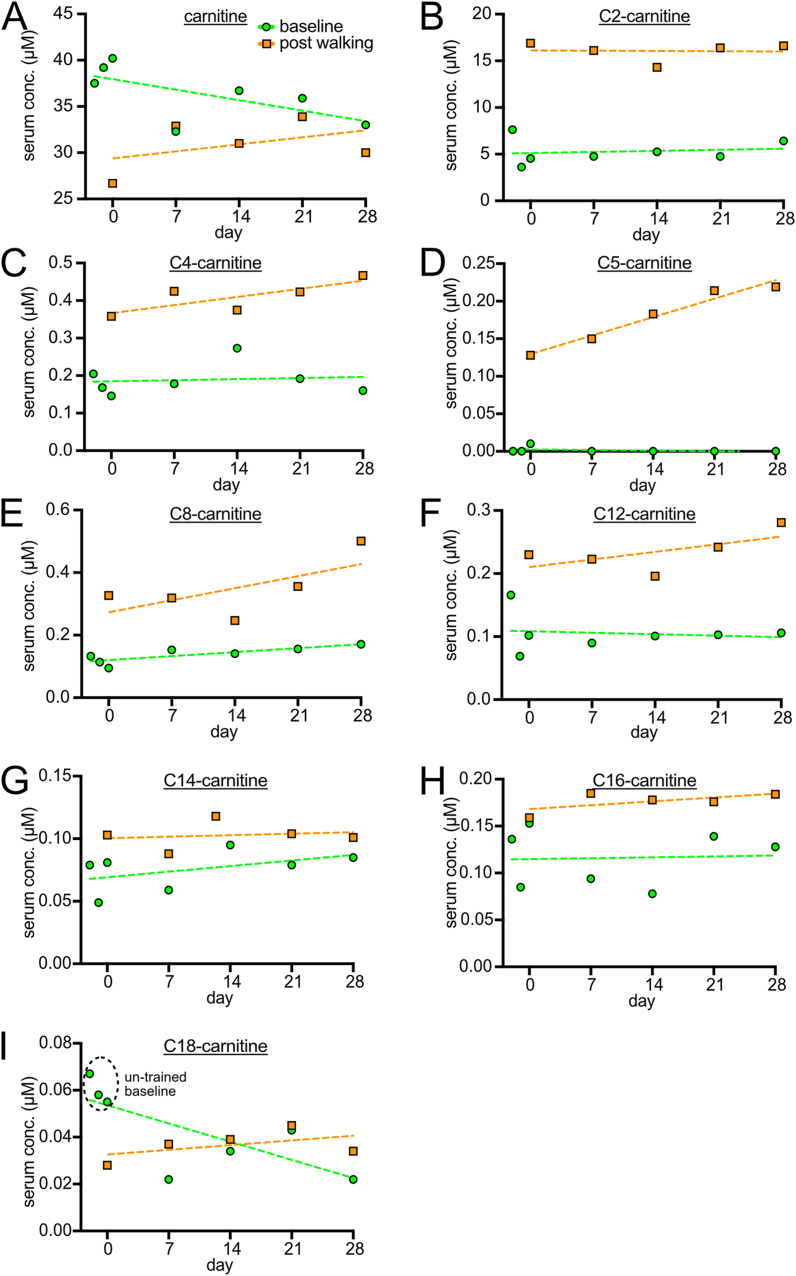
Serum Acylcarnitine levels (A–I) Levels of serum carnitine as well as acyl-carnitines over time. Baseline samples (green circles) were taken either prior to the start of the intervention (days −2, −1 or 0) or during the intervention (days 7, 14, 21 and 28), always 36 h after the last bout of walking. Post-walking samples (orange squares) were always taken directly after 200min of walking. C2-carnitine (B), C4-carnitine (C), C5-carnitine (D), C8-carnitine (E), and C12-carnitine (F) levels increase in post-walking samples and return to the stable baseline 36 h later. In comparison, C14-carnitine (G) and C16-carnitine (H) levels are only mildly increased in the post-walking samples. C18-carnitine levels (I) show an initial decrease at the first post-walking timepoint and maintain decreased levels in all following post-walking and trained baseline samples.

The metabolite with the most striking changes is C5-carnitine, which is detectable exclusively in post-walking samples and shows a strong training effect over the course of 4 weeks (Fig. 3D). By expanding the analysis to all other acylcarnitines (Fig. 3), it becomes clear that short-chain acylcarnitines (C2 to C5) show a significant increase in post-walking samples compared to baseline, and generally this trend becomes less pronounced as the acyl chain length increases (Fig. 3A–I). Since short- and medium-chain acylcarnitines are generated during fatty acid beta-oxidation from the breakdown of long-chain acylcarnitines, this pattern suggests that walking is strongly inducing beta-oxidation. Furthermore, training appears to increase the amount of beta oxidation, perhaps by increasing muscle mass. The beta-oxidation is entirely consistent with the substantial weight loss experienced by the subject (Fig. 1B). Combined with the fact that lactic acid levels did not increase (Fig. 1F) this suggests that walking stimulates aerobic metabolism more strongly than anaerobic metabolism. Baseline C18-carnitine, which is a major substrate of beta-oxidation, is used as a clinical diagnostic for beta-oxidation defects. When C18-carnitine levels are elevated, this indicates beta-oxidation is impaired [27,28]. Strikingly, we observed a drop in baseline C18-carnitine levels after the first walking intervention at day 0, and then C18-carnitine levels remain low, never reaching the un-trained baseline levels during the entirety of the intervention, neither in trained baseline samples nor in post-walking samples (Fig. 3I). No other metabolite displays a similar behavior. This suggests that the activation of beta-oxidation caused by walking is long-lasting.

### 3.4 Systemic succinate and aconitate increase hints at mitochondrial changes

**Fig. 4:**
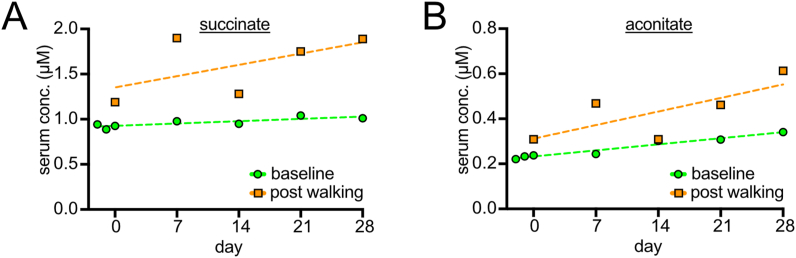
Mitochondrial metabolites. Succinate (A) and aconitate (B) levels increases in the post-walking samples in a training-dependent manner, but always drop again in the baseline samples. For aconitate, the baseline shows a statistically significantly increasing slope.

Both succinate (Fig. 4A) and aconitate (Fig. 4B) showed higher values post-walking, which further increase with training. Aconitate even shows a trend towards increasing in the trained baseline samples over time. The significance of these results is unclear. Since they are intermediates of the Krebs cycle, they may hint at systemic changes in mitochondrial metabolism. Alternatively, these metabolites could also originate from the gut microbiome. Circulating succinate has been shown to activate brown adipose tissue thermogenesis [29,30], which might contribute towards the elevated fatty acid beta oxidation we observe.

**Fig. 5:**
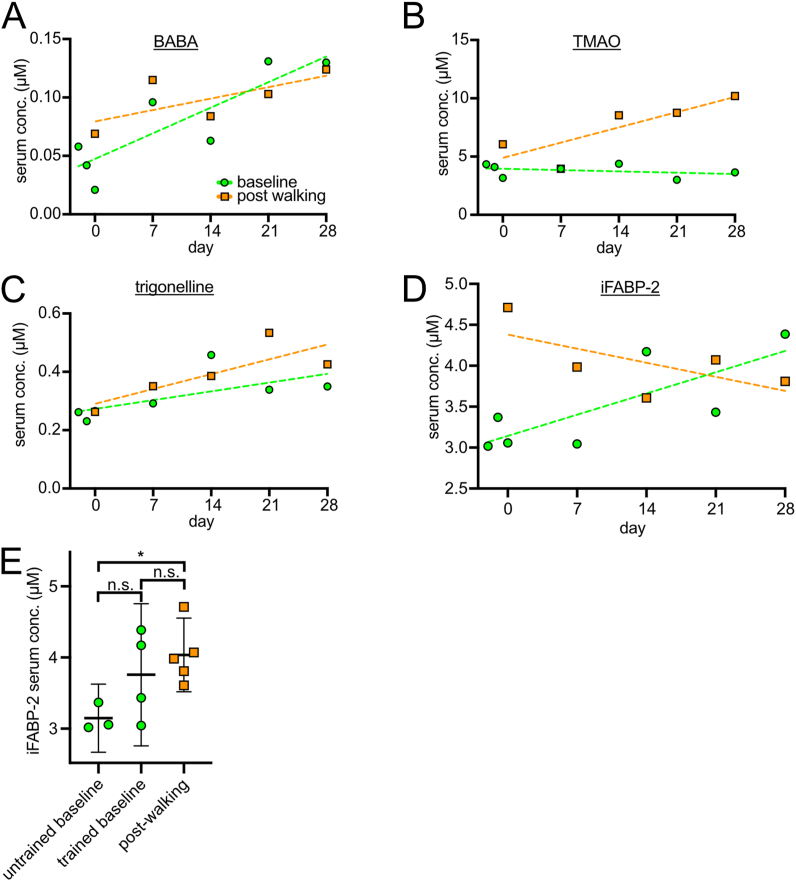
Metabolites which are derived from the gut microbiome (A) Beta-aminobutyrate (BABA) baseline and post-walking samples show an increase compared to untrained baseline samples. This increase correlates with training over time. (B) Trimethylamine N-oxide (TMAO) shows a training-dependent increase in the post-walking samples, while the baseline levels remain stable. (C) Trigonelline shows a training-dependent increase over time in the post-walking samples and a less pronounced increase in the trained baseline samples. (D) Baseline serum levels of iFABP-2, a protein from the intestinal lumen used as a readout for gut permeability, shows a positive trend that is not yet statistically significant when performing a correlation analysis of iFABP-2 levels and time (p = 0.17). (E) Serum concentration of iFABP-2 as measured by ELISA. Averages of technical duplicates are shown (error bars = 95% confidence interval). ∗p = 0.015 by 2-sided unpaired *t*-test.

### 3.5 Metabolites from the gut increase with training

One class of metabolites that significantly changes during the course of the intervention consists of metabolites that cannot be generated by human cells. The aminobutyrate BABA (beta-aminobutyrate, Fig. 5A) is structurally related to the human neurotransmitter GABA (gamma-aminobutyrate), but is part of the human ‘exposome’, predominantly found in cereals [31,32]. Trimethylamine N-oxide (TMAO) is a breakdown product of choline generated by the gut microbiome (Fig. 5B) [33]. Trigonelline is a product of niacin (vitamin B3) metabolism and can be found in some food sources [34] (Fig. 5C). Although trigonelline is abundant in coffee, coffee was not ingested at all by the subject during this study. With the exception of TMAO baseline levels, we noticed a general trend towards increasing levels of these gut metabolites during the course of the 4-week study. We do not know the significance of this, but one interpretation could be that the physical activity mildly increases gut permeability so that more gut metabolites can leak into the serum. To test this, we measured gut permeability using serum iFABP-2. This protein is expressed by gut enterocytes and secreted into the gut lumen. Presence of iFABP-2 in the blood indicates that gut luminal proteins can enter the blood stream. We found that indeed baseline levels of iFABP-2 increase over the course of the 4-week study (Fig. 5D and E), indicating mildly increased gut permeability that nonetheless is much below the level observed in pathological conditions [35]. The low constant baseline TMAO levels could be due to the fact that the baseline samples were all taken after overnight fasting and before breakfast, so that the gut might have been depleted of the TMAO precursor, choline. Although increased gut permeability could explain the progressive increase in exposome metabolites in the serum, an alternate explanation could be that the intervention modulated the composition of the gut microbiome so that it produces more of these metabolites, although this explanation seems less likely given that the various metabolites derive from different metabolic pathways.

## 4 Discussion

Our data suggest that walking can have a profound effect on the metabolism of a person – an effect that is visible both acutely as well as after 1.5 days. We designed the intervention to maximize weight loss while keeping the nutritional intake unchanged. We did not intervene to reduce caloric intake because calorie restriction can force the body to catabolize amino acid when energy demands increase during exercise, since glycogen storage is limited. This loss in muscle mass leads to a lower baseline energy expenditure after restrictive dieting [36,37], which often leads to the “yo-yo effect”, a weight gain up to, or even above, the starting weight shortly after returning to the previous eating habits [38,39]. During the course of our intervention the participant ate until satiety, as in the 6 months of sedentary lifestyle prior to the intervention. This was done with the intention to avoid muscle catabolism caused by caloric restriction.

In total we observed an astounding weight loss of 8.2 kg or 7.0% or −2.5 BMI units (kg/m^2^) in just 4 weeks of intervention, which is about half of what is observed in the first month after bariatric surgery [40]. It should be noted, however, that the rapid initial weight loss after bariatric surgery is due in part to dietary restrictions that are prescribed for optimal wound healing. For this reason, publications usually report follow-up data for an observation period starting three months after the surgery and lasting one or more years [41]. One such study showed a decrease in BMI of −6.0 kg/m^2^ in the roux-en-y gastric bypass group at one year of follow-up, or −0.5 kg/m^2^ per month on average [42]. Another intervention group of this study, which was treated with the weight-loss promoting antidiabetic drug exenatide showed a BMI decrease of −3.4 kg/m^2^ after one year [42], or −0.3 kg/m^2^ per month. Hence in comparison, the drop we observed of −2.5 kg/m^2^ in one month is quite large. We are limited in our comparison to bariatric surgery, however, as our intervention was shorter and ended before a plateau of either body weight or walking speed was reached.

The intervention described here was low-stress and aerobic, as shown by the stable cortisol and lactate levels, in contrast to other exerkinome screening approaches [44]. The acylcarnitine profile we observed suggests that walking increases fatty acid beta oxidation [45]. Other interventions have shown that low-intensity aerobic endurance training can lead to a higher total energy expenditure when compared to high-intensity training, which can only be performed for a short while due to fatigue [[46], [47], [48]], although high-intensity training can also have metabolic effects that extend into the recovery period. Not only the total energy expenditure is higher in longer-duration/lower-intensity exercise, but the contribution of beta oxidation as a highly oxygen-dependent energy source also increases [[49], [50], [51], [52]]. We utilized the fact that walking utilizes the largest muscle groups in the human body, which also consist of a majority of slow-twitch highly oxidative muscle fibers [53,54]. Therefore, walking can be considered the optimal physical activity to maximize fat loss via beta oxidation while minimizing fatigue, as muscles involved in standing upright have an inherently high endurance. Avoiding fatigue is key to being able to perform the activity for 200 min 6 days per week. In contrast to an exhaustive workout on a treadmill, walking on a deskmill can be done at home and in parallel to other activities, since the walking stride does not require focusing on the exercise and the arms remain free. Indeed, we picked the 200 min duration of the intervention as an approximation of the time an average German spends per day watching television [12], which can still be done uninterrupted while walking on a deskmill.

A sedentary lifestyle with low physical activity may mimic what other mammals, or our ancestors, experience during winter. This metabolic state of “hibernation” promotes a conservation of energy especially in the body’s fat stores [55], which is useful if animals have to survive a long winter with little nutrient availability. However, nowadays we are supplied with an abundance of food throughout the year, so a metabolic hibernation leads to a pathological accumulation of fat and therefore an epidemic of obesity. Our intervention which increases physical activity without reducing nutrient intake might be akin to the metabolic state of mammals in springtime, when both high activity and high nutrient availability promote muscle growth instead of conservation of fat mass [56]. From this point of view, caloric restriction may be counterproductive to ending the energy-conserving hibernation state.

With this in mind, we hypothesized that there could be a metabolic shift in the baseline metabolic state of the subject when comparing untrained to trained baseline samples taken after 36h of rest. Our results show that for most metabolites the baseline remained stable throughout the study. One of the few metabolites that showed a difference in baseline levels was C18-carnitine, which displayed a marked decrease at the first post-walking sample and never reached untrained baseline levels again. This finding is relevant because C18-carnitine is a substrate for mitochondrial beta-oxidation, because C18 has been shown to affect mitochondrial morphology [57] and because several other factors such as circulating succinate and aconitate hint at a metabolic adaptation on the mitochondrial level [28,[58], [59], [60]]. Hence, it is possible that mitochondrial activity is adjusted for a few days by the physical activity. Notably, elevated circulating succinate has been shown to activate brown adipose tissue thermogenesis, which might contribute to the elevated beta oxidative state we observed [29,30]. In mice, stimulation of SUCNR1 by succinate promoted a switch from fast-twitch to slow-twitch muscle fibers with higher mitochondrial content [61].

While our findings did not clearly identify a strong metabolic adaptation on the metabolome level in the baseline samples, the acute response to our low-intensity physical activity was more clear. The acylcarnitine profile suggests a boost in beta oxidation directly after walking [62,63], with C5-carnitine detectable only in post-walking samples and showing a marked training effect over time. While this metabolite has been shown to be elevated in heart failure [64], obesity and type 2 diabetes [65,66], it is important to consider that these pathological measurements were done in resting conditions, while in our experimental setup the resting baseline level of all acylcarnitines remained stable, except C18-carnitine, which decreased over time.

Surprisingly, some metabolites that showed a response to our intervention originate from the gut. These predominantly showed a mild increase over the timecourse of the intervention. This correlated with a mild increase in gut permeability, measured by quantifying levels of the gut luminal protein iFABP-2 in the serum. To our knowledge, this is the first time someone observes that a low-intensity physical activity influences iFABP-2 levels. This suggests that under this condition of prolonged physical activity the gut may become an endocrinologically active organ that releases or retains certain metabolites. The role of the gut in exercise has been described mostly under conditions of strenuous exercise, during which splanchnic ischemia can cause injury to the gut, eliciting diarrhea or even severe intestinal bleeding e.g. in marathon runners [35,67,68]. Research of these phenotypes showed that the presence of iFABP2 in the blood indicated a pathological leakiness of the gut which increases drastically in a hypoperfused gut setting, e.g. during gut surgery [69] or acute intestinal ischemia [70]. As the cutoff values that differentiate normal from pathological iFABP-2 levels are strongly dependent on the sensitivity of the assay used, there is no established reference range. Therefore, looking at the intraindividual change is considered a more reliable method of assessing gut leakiness [35]. In our iFABP-2 ELISA we detected an increase of only 1.3-fold on average, less than reported in another study, which showed an increase of at least 2-fold, albeit at a much higher exercise intensity [71]. That study showed no translocation of bacteria to the bloodstream, highlighting the possible physiological role of “gut leakiness” that seems to be different from a pathological leakiness from ischemia, surgery or gut injury in athletes. Our findings support this notion, as our experimental settings avoided strenuous exercise in favor of a more physiological increase in physical activity. In addition to changes in gut leakiness, there may also be some changes in the gut microbiome composition induced by the physical activity, as has been reported previously for exercise [[72], [73], [74], [75]]. We did not collect stool samples, however, to screen for changes in composition of the microbiome because we did not expect to see changes in metabolites originating from the microbiome. Furthermore, understanding how changes in the intestinal microbiome affect intestinal metabolites can be complex and will require further study. Many exerkines are grouped under their tissue of origin such as hepatokines (liver) or batokines (brown adipose tissue). We propose the term “enterokines” to describe exerkines originating from the gut or its contents.

Several of the gut metabolites we detect have been linked to diseases. TMAO, which results from a breakdown of choline in the gut, was described as a marker for heart failure [33,71,76]. Of note, the studies that found the correlation between TMAO and heart failure in patients investigated baseline samples [77], while in our experimental setup the baseline remained constant, which hints at our intervention not being harmful to the heart. Yet, the post-walking levels were elevated and continued to be even more elevated with training. One could hypothesize that for patients with heart failure daily activities like walking to the study center may already constitute exercise so their TMAO levels would be higher than control patients. This could suggest that TMAO levels are actually a correlate of physical activity and are not actually harmful to the heart itself. Indeed, TMAO levels have previously been reported to be elevated in healthy athletes [74]. Trigonelline has been shown to reduce proteinuria and to protect renal function in diabetic nephropathy [78,79]. Despite being part of the human exposome, our data show a training-dependent increase in both trained baseline and post-walking samples.

### 4.1 Limitations of the study

One clear limitation of our study is that the intervention was performed on a single subject. Hence it remains to be tested whether similar weight loss can be achieved on a larger cohort of people. The results obtained here provide the basis for designing such a future study. Furthermore, the ursodeoxycholic acid given to the subject could be a confounder, although previous studies have shown that ursodeoxycholic acid does not cause weight loss [43]. Finally, we did not control for changes in calorie intake – we simply told the subject to continue eating normally, and asked the subject weekly that this was indeed the case. Hence it is possible that the subject may have reduced food intake somewhat, in which case the weight loss reported here over-estimates the effect of the walking *per se*. Alternatively, the subject may have increased food intake due to the increased physical activity, in which case the weight loss reported here actually underestimates the effect of the walking alone. Nonetheless, this regimen mimics what would happen if we ask subjects to walk daily without applying a dietary intervention.

## 5 Conclusion

In conclusion, this feasibility study shows that an intervention of 200 min of walking per day has a profound effect on the metabolic profile of a person. It leads to a substantial drop in body weight, similar in magnitude to that seen after bariatric surgery, and activates fatty acid beta-oxidation. It will be interesting to use the reference datapoints we describe here to study these parameters on a larger cohort of people in the future.

## Ethics statement

The study design was approved by the local ethics committee (Ethics Committee of the Medical Faculty of Heidelberg: Trial Code Number S-518/2022).

## Author contribution statement

Daniel Pfaff and Gernot Poschet: Conceived and designed the experiments; Performed the experiments; Analyzed and interpreted the data; Contributed reagents, materials, analysis tools or data; Wrote the paper.

Rüdiger Hell and Julia Szendrödi: Conceived and designed the experiments; Analyzed and interpreted the data; Wrote the paper.

Aurelio Teleman: Conceived and designed the experiments; Analyzed and interpreted the data; Contributed reagents, materials, analysis tools or data; Wrote the paper.

## Data availability statement

Data included in article/supp. material/referenced in article.

## Declaration of competing interest

The authors declare that they have no known competing financial interests or personal relationships that could have appeared to influence the work reported in this paper.

## Appendix A Supplementary data

Multimedia component 5 Multimedia component 5

figs1 figs1
