# Supplementary figures and images for: Walking 200 min per day keeps the bariatric surgeon away
Source: Heliyon. 2023 May 22;9(6):e16556. doi: 10.1016/j.heliyon.2023.e16556 (PMC10238728; doi:10.1016/j.heliyon.2023.e16556)

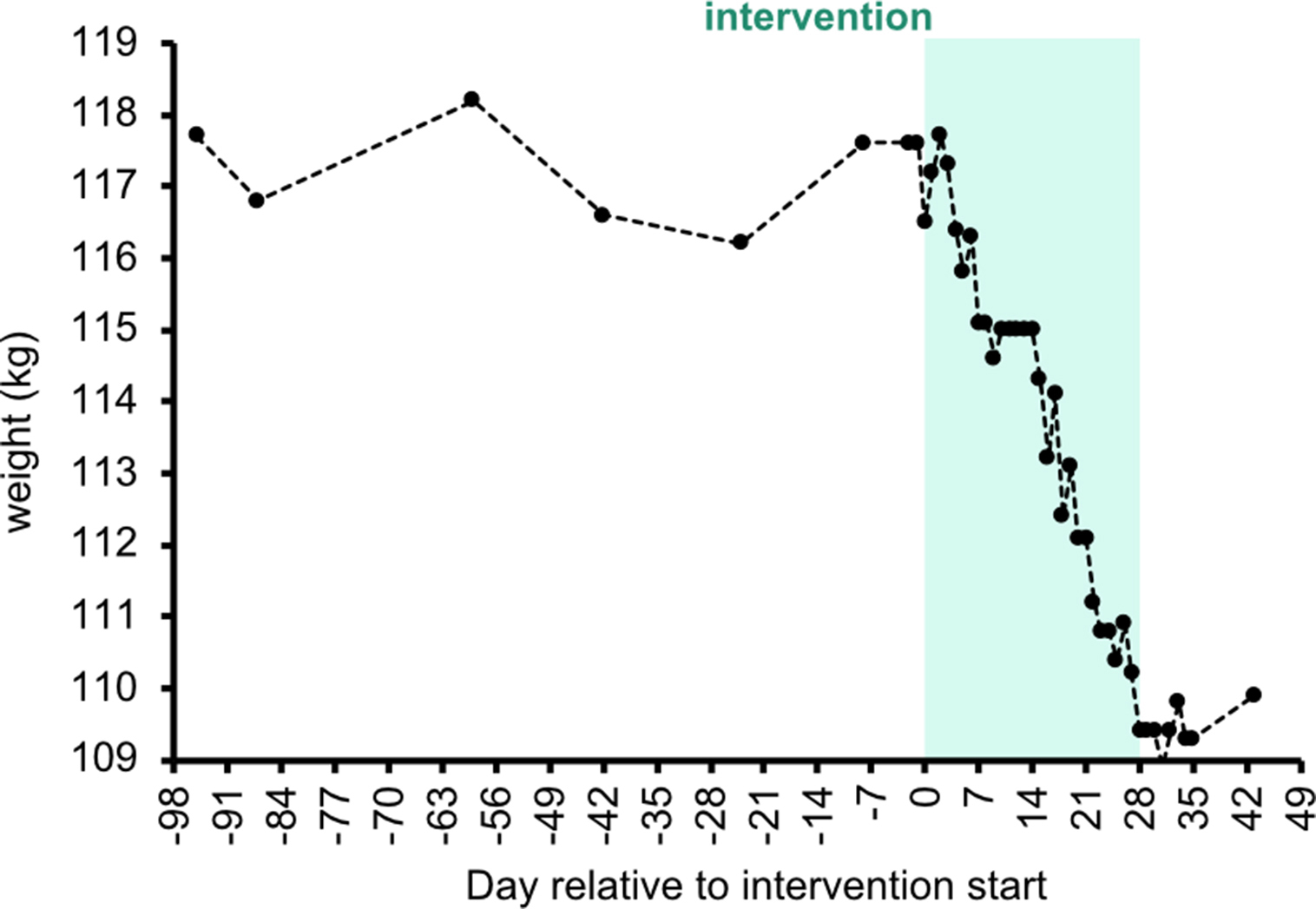

Supplement: figs1 [file mmcfigs1.jpg]
